# Green Synthesis of Silica Nanoparticles from Sugarcane Bagasse Ash for Stable Pickering Oil-in-Water Emulsions

**DOI:** 10.3390/molecules30224464

**Published:** 2025-11-19

**Authors:** Daniel Jaramillo-Vélez, Mariana Ochoa-Castaño, Andrea Flórez-Caro, Luis David Botero, Esteban Ureña-Benavides, Raúl Adolfo Valencia-Cardona, Jorge Andrés Velásquez-Cock, Catalina Gómez-Hoyos

**Affiliations:** 1 Programa de Ingeniería en Nanotecnología, Universidad Pontificia Bolivariana, Circular 1—No 70-01, Medellín 050031, Colombia; 2 Department of Biomedical Engineering and Chemical Engineering, The University of Texas at San Antonio, One UTSA Circle, San Antonio, TX 78249, USA

**Keywords:** silica nanoparticles, silicon dioxide nanoparticles, Pickering emulsions, green synthesis, sugarcane bagasse, oleic acid, adsorption modification, stability, agro-industrial by-product, sustainable materials

## Abstract

The present study explores novel alternatives for the exploitation of sugarcane bagasse ash by obtaining and modifying SiO_2_ nanoparticles through a green synthesis method. The hydrophilic nature of the nanoparticles was modified using oleic acid. The nanoparticles were characterized using FTIR, FESEM, and DLS, and their performance in the stabilization of Pickering emulsions was also studied. FESEM micrographs of the nanoparticles revealed an irregular and agglomerated structure. EDS confirmed that their main components are oxygen and silicon, and ATR-FTIR spectra demonstrated that oleic acid effectively modified the nanoparticles. Subsequently, O/W Pickering emulsions were fabricated by combining rotor–stator homogenization and probe ultra-sonication, using dodecane and liquid paraffin as model oil phases and SiO_2_ NPs as stabilizers. Static light scattering measurements showed that the emulsions exhibited polydispersity, while photographic monitoring confirmed that their physical stability was affected by the concentrations of oleic acid and nanoparticles: concentrations of up to 20.0 wt% and 1.0 wt%, respectively, produced emulsions that remained stable for 7 to 15 days. This study identifies the behavior and challenges associated with novel pathways for the valorization of sugarcane bagasse ash. The stabilization of Pickering emulsions using the obtained SiO_2_ NPs highlights their potential in pharmaceutical, cosmetic, and food applications.

## 1. Introduction

Sugarcane is one of the most important crops worldwide, with annual production exceeding 1.9 billion tons in 2023 [1]. It is processed by the mechanical extraction of sugarcane juice, which is further transformed to obtain refined sugar or to produce bioethanol; during this process, different by-products are generated [2]. Among them, sugarcane bagasse, the fibrous material derived after the sugarcane juice extraction, is an interesting material, as it is rich in hemicelluloses, cellulose, lignin, and minerals [3]. It is estimated that nearly 250 kg of sugarcane bagasse is generated for each ton of sugarcane processed [4].

Besides its potential as a raw material of different biomolecules, sugarcane bagasse is commonly used as a biofuel in traditional furnaces, such as those used in the processing of panela or piloncillo, a sweetening alternative derived from sugarcane; it is typical of Latin America and produced mostly by small communities [5]. After combustion, 1 ton of bagasse is reduced to 25 kg of sugarcane bagasse ash (SBA), which is rich in minerals, mainly silica, with a content of up to 96.93 wt.%, depending on soil composition and plant growth [6]. This ash has been traditionally used as fertilizer; however, its mineral profile and low concentration of nutrients require its mixing with other compounds before its application, highlighting the need for different studies into its exploitation. These reports have focused on its potential to reinforce cementitious materials [6,7,8]; nevertheless, they overlook other alternatives for this by-product.

Its silica content has prompted its evaluation as a raw material to produce silica-based ordered mesoporous material [9] and silica nanoparticles (SiO_2_ NPs) [3]; these nanoparticles consist of a colloidal dispersion of SiO_2_ particles with ranges from 1 to 100 nm, and they are usually synthesized by the Störber method, which uses a silica precursor, such as Tetraethyl Orthosilicate (TEOS), and a basic catalyzer, such as NH_4_OH [3,10]. The development of different pathways using renewable sources is of interest as it helps to strengthen the productive chain of sugarcane and provides alternative high-value applications where small communities could access as raw material providers for the obtention of SiO_2_ NPs, a material with an estimated market value of 8.8 billion USD in 2020 [3,10]. Several studies have reported the inhalation toxicity [11] and nephrotoxicity [12] of TEOS. Consequently, the development of different pathways using renewable sources as silica precursor is of interest as it helps to strengthen the productive chain of sugarcane and provides alternative high-value applications where small communities could access as raw material providers for the obtention of SiO_2_ NPs.

The valorization of sugarcane bagasse ash through the synthesis of SiO_2_ NPs represents not only an environmentally friendly alternative but also an opportunity to strengthen and diversify the sugarcane production chain through technological development. In this context, hard technologies [13], such as nanomaterials synthesis, and soft technologies, which aim to implement biorefinery models that integrate traditional production with the development of new inputs for emerging sectors through knowledge management [13], play a key role in innovation and sustainability within agro-industrial systems. The sugarcane sector, traditionally focused on sugar and energy production, still faces technological gaps that limit its diversification and the integral valorization of its by-products. The incorporation of nanotechnology in this production chain contributes to closing these gaps, promoting technology transfer, the generation of new business models based on circular economy principles [14] and fostering research and institutional collaboration, as well as increasing the economic value of this by-product [15,16]. This approach contributes to achieving the Sustainable Development Goals (SDG 9, 12 and 13) by encouraging sustainable innovation, efficient resource management and reduction in environmental impacts associated with sugarcane production [17].

Nevertheless, the synthesis of SiO_2_ NPs comes with important challenges: the intended application impacts the synthesis and adsorption modification of these nanoparticles, as changes in the nanoparticle surface can affect their performance [10]. Among the application of these nanoparticles, their use as an emulsifier is of interest for agricultural and food industries, where there is mounting pressure to reduce the consumption of synthetic emulsifiers, such as surfactants, in different formulations, resulting in a cleaner label [18]. In this context, the use of solid-stabilized emulsions, also known as Pickering emulsions, is an important and evolving field of application for SiO_2_ NPs [19].

Pickering emulsions are obtained when a solid particle is adsorbed at the oil/water interphase at the edge of the droplets [19,20]. The energy required to reverse this adsorption depends on particle size and the contact angle of the particle at the O/W interphase, θ, while a theoretical maximum is observed at 90 °; however, this value shall be a little lower for O/W emulsions and a little higher for W/O, allowing for the particle to move towards the continuous phase, while both phases wet the particle surface [20]. SiO_2_ NPs are traditionally hydrophilic, allowing the unmodified particles to stabilize emulsions with polar oils while reducing their ability to stabilize non-polar phases [19,21]. Therefore, the properties of the synthesized SiO_2_ NPs shall be tailored to satisfy these requirements and stabilize the resultant emulsions. This implies that the nanoparticles shall be modified.

There are different strategies to achieve this modification, while traditional methods rely on chemical functionalization and the formation of covalent bonds of non-polar aliphatic chains to a silane atom; compounds as polyethylene glycol silanes [21] have been hydrolyzed and reacted with unmodified SiO_2_ NPs. Novel techniques, aiming to reduce the reliance on chemical synthesis, have been proposed, achieving the adsorption of different emulsifiers, such as cetyl trimethyl ammonium, sodium dodecyl sulfate [22], and oleic acid [23], among other compounds. Oleic acid has been reported as an interesting option as it is a biodegradable, biocompatible fatty acid and has been used as an emulsifier in water in oil emulsions [24], appearing as a sustainable alternative to synthetic compounds, showing interesting results in promoting the stabilization of Pickering emulsions with different oils. Nevertheless, most of these works evaluate its adsorption over synthetic monodisperse SiO_2_ NPs [22,25,26] and rice paddy husk [23]. To the authors knowledge, there is no exploration of the effect of this type of adsorption modification using nanoparticles derived from sugarcane bagasse ash using green methods, which might result in a wide arrange of nanoparticle distribution, thus hindering the exploration of these nanoparticles in food and agricultural applications.

This work aims to provide novel alternatives to exploit sugarcane bagasse ash by synthesizing and modifying SiO_2_ NPs using green methods and evaluating their physical and morphological characteristics by attenuated total reflection–Fourier transform infrared spectroscopy (ATR–FTIR), field emission scanning electron microscopy and energy-dispersive X-ray spectroscopy (FESEM-EDS) and static light scattering (SLS) analysis, as well as their performance in the formation of Pickering emulsions using two model oils, dodecane and paraffin, assessing their stability by physical observation, in order to identify the effect of nanoparticle and oleic acid concentration in emulsion formation and stability. We aim to identify the behavior and challenges of novel pathways of sugarcane bagasse ash valorization.

## 2. Results

### 2.1. Nanoparticle Characterization

#### 2.1.1. Field Emission Scanning Electron Microscopy (FESEM)

**Figure 1:**
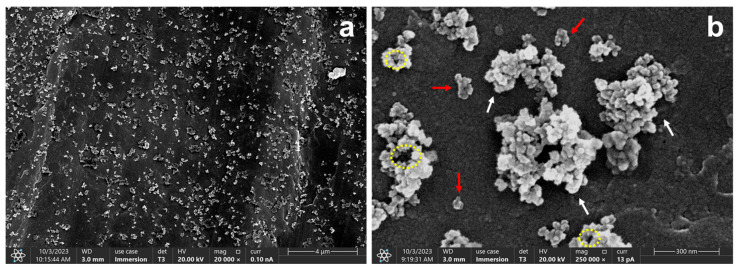
FESEM micrographs of the unmodified nanoparticles at (**a**) 20,000× and (**b**) 250,000×. Red arrows indicate the presence of smaller or less aggregated particles, while white arrows represent larger clusters. Yellow dotted circles indicate the presence of micropores.

Morphological and compositional characterization were carried out through FESEM and EDS analyses of the obtained SiO_2_ NPs. Figure 1a,b show the presence of particle agglomerations of irregular morphologies, which is explained by the presence of micropores between silica grains, highlighted by yellow dotted circles in Figure 1b [27]. This type of conformation is favored by the destabilization of the three-dimensional network formed by the NPs during the drying process, which results in particle agglomeration and the formation of a xerogel [4].

A mean Feret diameter of 526.36 ± 262.36 nm was obtained, indicating a broad size distribution. This large variability can be attributed to the coexistence of both isolated nanoparticles and agglomerated structures formed during sample preparation. The isolated nanoparticles exhibited Feret diameters in the range of 43.60–120.54 nm, whereas larger particles, corresponding to aggregated structures or clusters, showed sizes between 146.90 and 603.10 nm.

**Figure 2:**
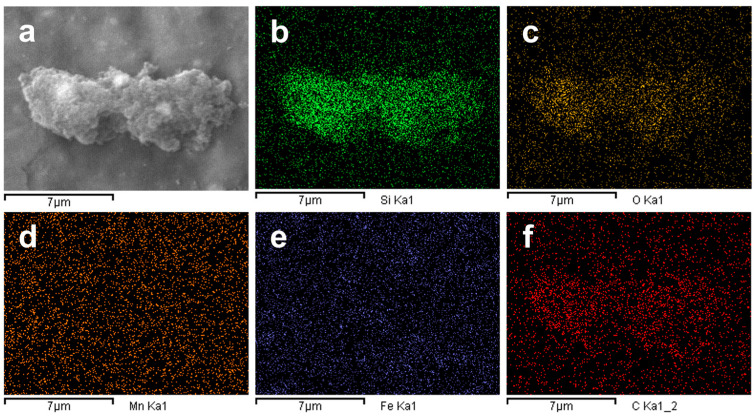
EDS compositional mapping of nanoparticles: (**a**) FESEM-EDS micrograph; (**b**) silicon (Si) mapping; (**c**) oxygen (O) mapping; (**d**) manganese (Mn) mapping; (**e**) iron (Fe) mapping; (**f**) carbon (C) mapping.

Additionally, EDS analysis, Figure 2, indicates that samples are mainly composed of silicon and oxygen, with traces of elements such as carbon, iron and manganese, mostly on the background of the pictures which could come from the sample holder, or traces of dissolved ions. Results obtained in both FESEM and EDS analyses are consistent whit previous reports that produced silicon nanoparticles from sugarcane bagasse and SBA, such as the work of Falk et al. [4] and Rovani et al. [28]. They obtained a similar microstructure and heterogeneous morphology with the presence of agglomeration of nanoparticles, as well as an elemental composition that included the presence of impurities such as carbon, iron, aluminum, manganese and sodium [4,27,28,29,30].

In summary, these particles present an irregular, agglomerated structure, mainly composed of oxygen and silicon. According to these results, the green synthesis of silica nanoparticles provides a pathway for nanoparticle production, which reduces the need for the reagents used in conventional synthesis processes [3,31], and it increases the value of agricultural byproducts, contributing to a more efficient use of resources. Furthermore, the synthesis method described in this study utilizes only HCl and NaOH as reagents and demonstrates the possibility of obtaining nanoparticles composed primarily of oxygen and silicon.

#### 2.1.2. Attenuated Total Reflection–Fourier Transform Infrared Spectroscopy (ATR–FTIR)

While EDS made it possible to identify that the main elements present in the synthesized material are oxygen and silicon, ATR-FTIR was used to verify that these elements formed chemical bonds characteristic of silica nanoparticles [23,25,30].

**Figure 3:**
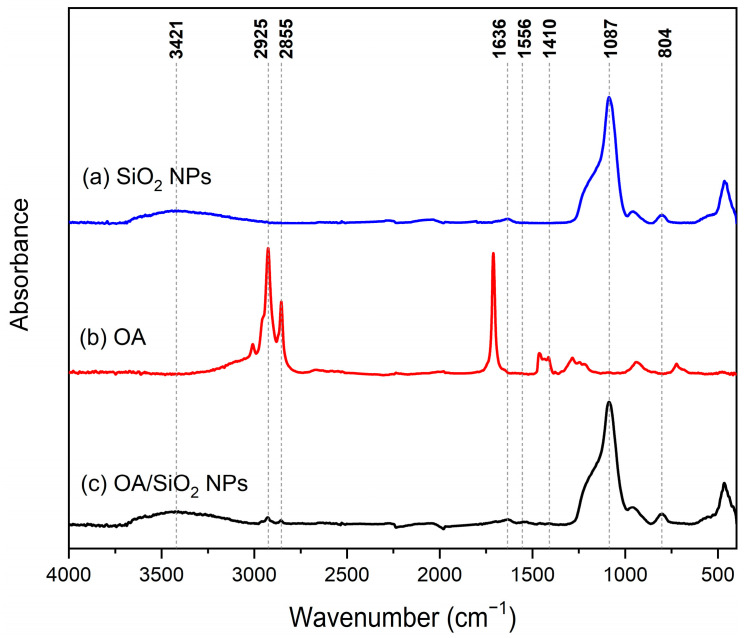
FTIR spectra of (**a**) unmodified SiO_2_ NPs; (**b**) pure oleic acid, (**c**) SiO_2_ NPs modified with oleic acid following the nanoparticle/oleic acid ratio of sample P-HH.

FTIR spectra, shown in Figure 3a reveals distinctive characteristics of nanoparticles obtained through green synthesis methods. According to Ni’mah et al. [30], absorption peaks in the region of 3421 cm^−1^ and 1636 cm^−1^ correspond to the stretching vibration and bending vibration, respectively, of –OH and H_2_O silanol groups present in silica nanoparticles, indicating their formation from sugarcane bagasse ash. Additionally, the characteristic peaks at 1087 cm^−1^ and 804 cm^−1^ are attributed to the asymmetric and symmetric stretching vibration of the Si-O-Si bond, respectively, confirming the silica structure. This evidence suggests the synthesis of silica nanoparticles through the selected approach [30].

However, characterization carried out through FESEM and EDS analyses revealed agglomeration of nanoparticles, indicating the need for their modification to enhance sol stability. The need for adsorption modification extends to improve the stability of the developed Pickering emulsions. Similarly, a literature review shows that silica nanoparticles are highly hydrophilic in character [22,23,32]. Therefore, an adsorption modification strategy to enhance the lipophilic character of the nanoparticles must be developed to improve their performance in the formulation of Pickering emulsions.

ATR-FTIR was used as well, to evaluate silica nanoparticles before and after their modification with 20 wt.% oleic acid (based on nanoparticle weight), comparing the results with the spectrum of pure oleic acid. ATR-FTIR spectroscopic analysis provides insights into the modification of silica nanoparticles with oleic acid and the significance of this modification in Pickering emulsion stabilization.

FTIR spectrum of oleic acid is presented in Figure 3b. Two distinct peaks are evident at 2853 cm^−1^ and 2921 cm^−1^, corresponding to symmetric and asymmetric stretching vibrations of –CH_2_ groups, respectively [23]. Additionally, a moderately sharp peak at 3005 cm^−1^, overlapping with the O–H stretching band, is attributed to C–H stretching within the vinyl moiety [23]. Furthermore, a sharp peak appearing at 1714 cm^−1^ predominantly signifies C=O stretching, while the peak around 1285 cm^−1^ is associated with C–O stretching within the carboxylic group [23].

FTIR analysis reveals significant changes between the spectra of silica nanoparticles modified with oleic acid and the unmodified nanoparticles (see Figure 3a,c). Premaratne et al. noted that the characteristic bands at 1093 cm^−1^, 800 cm^−1^, and 460 cm^−1^ correspond to the Si-O-Si vibration modes, which persist in both modified and unmodified nanoparticles [23]. The absorption peaks at 2855 and 2925 cm^−1^ depicted in Figure 3b,c are indicative of stretching vibrations specific to the –CH_2_ group [23,25], which are absent in the spectrum of pure silica nanoparticles shown in Figure 3a. This observation suggests the presence of long alkyl chains within the modified SiO_2_ NPs incorporating oleic acid. These results confirm the successful modification of silica nanoparticles, which is crucial for the effective stabilization of Pickering emulsions [23,30].

### 2.2. Emulsion Characterization

#### 2.2.1. Physical Stability

**Figure 4:**
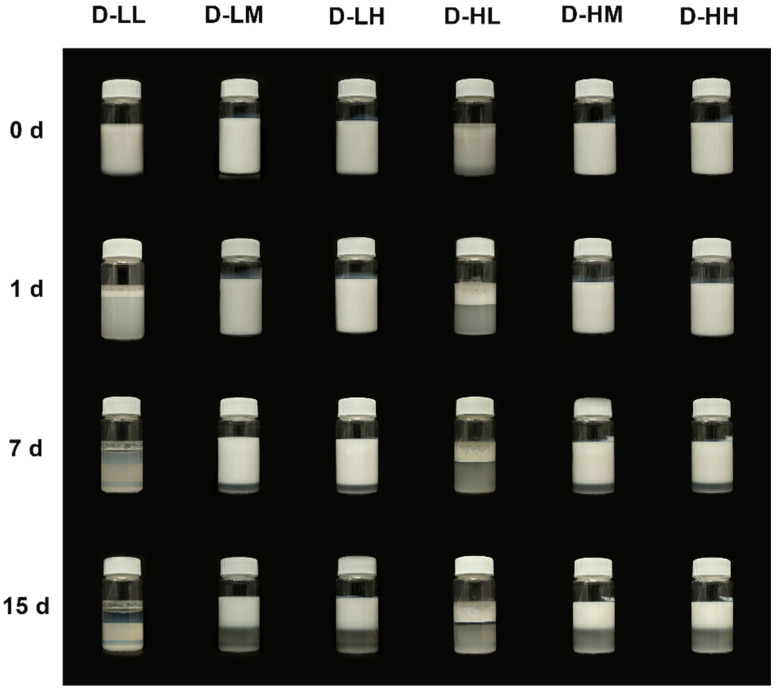
Physical stability of different formulations of dodecane-water emulsions stabilized with SiO_2_ NPs modified with adsorbed OA, where: LL refers to low concentration of SiO_2_ NPs and low concentration of OA; LM refers to low concentration of SiO_2_ NPs and medium concentration of OA; LH refers to low concentration of SiO_2_ NPs and high concentration of OA; HL refers to high concentration of SiO_2_ NPs and low concentration of OA; HM refers to high concentration of SiO_2_ NPs and medium concentration of OA; and HH refers to high concentration of SiO_2_ NPs and high concentration of OA. Pictures were taken after immediately after emulsification (0 h), 1 day after (1 d), 7 days (7 d) and 15 days (15 d).

**Figure 5:**
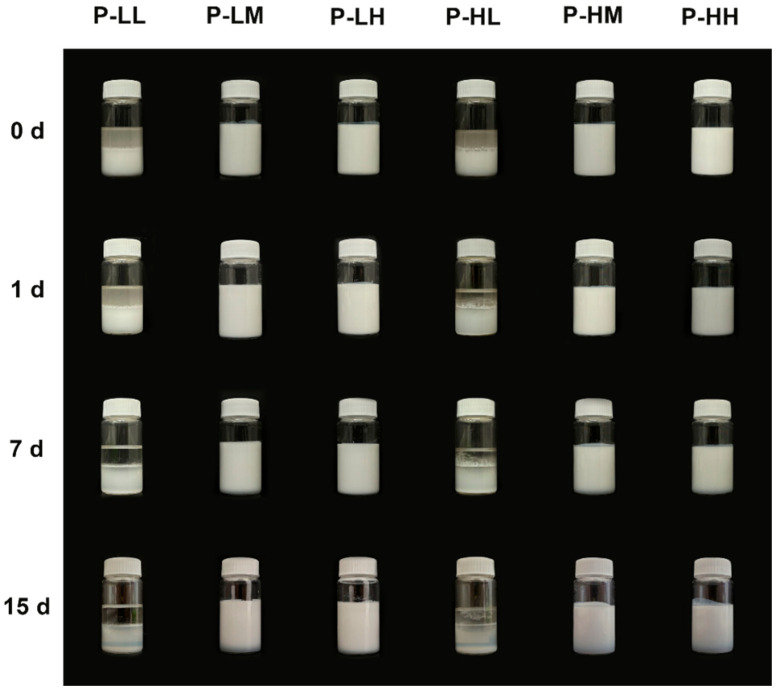
Physical stability of different formulations of liquid paraffin-water emulsions stabilized with SiO_2_ NPs modified with adsorbed OA, where: LL refers to low concentration of SiO_2_ NPs and low concentration of OA; LM refers to low concentration of SiO_2_ NPs and medium concentration of OA; LH refers to low concentration of SiO_2_ NPs and high concentration of OA; HL refers to high concentration of SiO_2_ NPs and low concentration of OA; HM refers to high concentration of SiO_2_ NPs and medium concentration of OA; and HH refers to high concentration of SiO_2_ NPs and high concentration of OA. Pictures were taken after immediately after emulsification (0 h), 1 day after (1 d), 7 days (7 d) and 15 days (15 d).

Multiple factors can influence the stability of emulsions; therefore, analyzing their behavior over time is crucial in its understanding. In the present article, a photographic record was used to evaluate the qualitative stability of the obtained emulsions. Photographs of the physical stability test of dodecane and liquid paraffin emulsions are presented in Figure 4 and Figure 5. For both types of emulsions, it is observed that those prepared with low concentrations of nanoparticles and/or devoid of oleic acid, destabilized immediately after preparation. In contrast, emulsions using dodecane prepared with medium and high concentrations of SiO_2_ NPs modified with adsorbed oleic acid remained stable for up to 7 d, with minimal creaming, and those prepared with liquid paraffin PL-H (0.5% SiO_2_ NPs and 20 wt.% oleic acid) remained stable for up to 15 d with no evident creaming. To confirm the effect of the SiO_2_ NPs, the stabilization of dodecane using pure oleic acid was also tested, resulting in a mixture that did not form a stable emulsion, as can be seen in Figure S1. Oleic acid is insoluble in water and has an HLB of 1, being used in stabilizing water in oil emulsions [33], while the stabilization of oil-in-water emulsions with oleic acid is frequently achieved with the addition of cosurfactants, such as polyoxyethylene sorbitan monolaurate (Tween 20) [34]. This result is coherent with the findings of Sadeghpour et al. [22], who reported that oleic acid shows a very low stabilizing effect for O/W emulsions due to its very low hydrophilic-lipophilic balance value. To summarize, the influence of nanoparticle concentration, their adsorption modification, and type of oil phase used on emulsion stability is evident, while none of the materials alone could achieve a stable emulsion. Further characterizations were only performed in samples that remained stable for more than one day.

As previously mentioned, silica nanoparticles alone do not effectively stabilize emulsions due to their high hydrophilicity, hindering their adsorption at the water/oil interface [35]. The adsorption modification of silica nanoparticles with oleic acid results in a significant increase in emulsion stability, demonstrating the synergism between the nanoparticles and oleic acid in stabilizing the emulsions, preventing phase separation even after 15 d in the case of emulsions prepared with liquid paraffin, which showed greater stability. Similar results were reported by Lin et al. [36], who synthesized Fe_3_O_4_ coated with oleic acid to first produce W/O Pickering emulsions and used the modified particles to stabilize W1/O/W2 emulsions. Sadeghpour et al. [22] also fabricated emulsions with a similar behavior, modifying hydrophilic silica nanoparticles using oleic acid to produce O/W Pickering emulsions; the modified silica nanoparticles enabled the stabilization of emulsions containing various oil phases, such as long-chain alkanes and liquid paraffin.

Therefore, the modification of silica nanoparticles with oleic acid is crucial to achieve Pickering emulsions that remain stable for longer periods. The literature suggests that the presence of long alkyl chains in the modified nanoparticles allows for a more hydrophobic nanoparticles, with organic chains surrounding the surface of silica NPs [37], promoting their interaction with the oil phase of the emulsion, thereby facilitating its stabilization. In other words, oleic acid reduces the hydrophilic behavior of silica nanoparticles, enhancing their compatibility with organic solvents and oil [22,23,25,36,38].

On the other hand, the enhanced stability to creaming of liquid paraffin emulsions compared to those utilizing dodecane can be elucidated through Stokes’ Law (Equation (3)) [39], a mathematical model that represents the gravitational separation rate of spherical droplets.

(1) $$V_{Stokes}=\frac{2gr^{2}\left|\rho_{2}-\rho_{1}\right|}{9\eta_{1}},$$

$V_{Stokes}$ denotes the gravitational separation rate, with g as the gravitational constant, r representing the droplet radius, and $\rho_{1}$ and $\rho_{2}$ the densities of the continuous and dispersed phases, respectively. A higher $\rho_{2}$ value, while being lower than $\rho_{1}$, results in a reduced $\left|\rho_{2}-\rho_{1}\right|$ value alongside $V_{Stokes}$. Essentially, owing to greater density of liquid paraffin (0.880 g/cm^3^) relative to dodecane (0.753 g/cm^3^), it exhibits a diminished gravitational separation rate, thereby improving its stability. Multiple studies have proved the correlation between increased oil density and enhanced emulsion stability [40,41,42].

#### 2.2.2. Emulsification Index

To quantitatively rectify the aforementioned observations, the emulsification index was calculated. The emulsification index (EI) is a measure of the emulsion’s stability during storage and primarily indicates the emulsion’s capacity to resist flocculation and sedimentation caused by gravity [43]. In other words, the EI increments alongside the emulsion stability, with an EI of 100% indicating a completely emulsified system. The EI was calculated for all samples that remained stable longer than 1 day; samples D-LL, D-HL, P-LL, and P-HL destabilized right after fabrication, thus not producing relevant data. The results are shown in Table 1.

Firstly, for measurements taken in 1 d, samples do not present statistically different EI, with all values being close to 100%. Nonetheless, samples at 15 d exhibit significantly different behavior between oil phases; dodecane samples present lower EI values, ranging from 58.296% ± 1.794% to 66.858% ± 10.985%, whereas liquid paraffin samples present significantly higher indexes, ranging from 88.638% ± 0.639% to 93.998% ± 0.923%. As previously mentioned, this can be explained by the difference in density between both oil phases, where the higher density of liquid paraffin is associated with a diminished gravitational separation rate. Furthermore, sample P-HH is particularly interesting, as its EI does not change significantly between 1 d and 15 d, which can be associated with a higher content of both nanoparticles and oleic acid, thus resulting in a higher stability. Calculations of EI for 0 d and 7 d are available in the Supplementary Information (Table S1).

**Figure 6:**
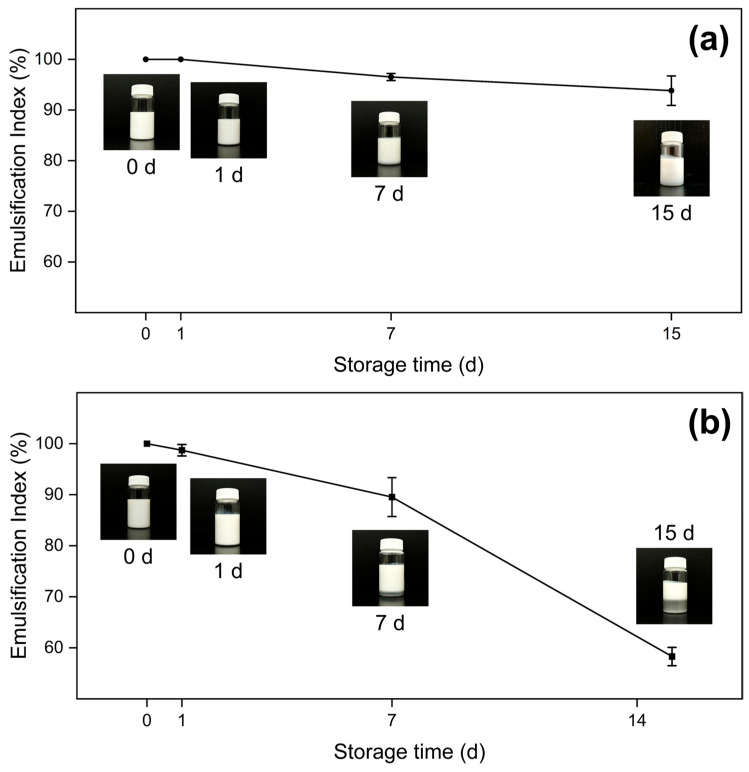
Emulsification index over time for the samples (**a**) P-HH, and (**b**) D-HH.

Figure 6 shows representative samples for the EI behavior of both oil phases; Figure S2 presents data for the rest of the samples. Paraffin samples’ EI decrease slower over time compared with dodecane’s, as depicted in the inset photographs: the P-HH 15 d (Figure 6a) sample remains mostly uniform, while the D-HH 15 d (Figure 6b) sample shows two distinct phases.

#### 2.2.3. Particle Size Distribution

**Figure 7:**
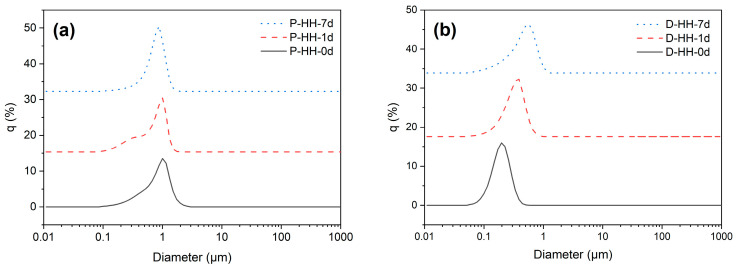
Size distribution at different time stamps: after emulsification (0 d), 1 day after (1 d) and 7 days after (7 d), for emulsions with (**a**) paraffin for oil phase and (**b**) dodecane for oil phase.

Another parameter to consider when characterizing emulsions is droplet size [44]. Only the most stable emulsions for each oil phase were analyzed, considering the previous stability analysis, namely P-HH and D-HH. Figure 7a,b show the size distributions of paraffin and dodecane emulsions, respectively, measured at three times: immediately after preparation (0 d), after one day (1 d), and after one week (7 d).

Table 2 shows the d_10_, d_50_, d_90_, D_4,3_, and δ values obtained for these samples. The values of d_10_, d_50_, and d_90_ were used to calculate δ as indicated in Table 2. For all analyzed samples, both paraffin and dodecane, it is evident that δ is greater than 0.5, indicating polydispersity in droplet sizes [44]. Overall, emulsions with a dodecane oil phase tend to show lower δ and, therefore, lower polydispersity [44].

The mean particle diameter (D_4,3_) was used to monitor the stability of the emulsions and their aggregation [40,45]. Dodecane emulsions showed a greater increase in diameter (from 0.181 ± 0.007 μm to 0.558 ± 0.039 μm) compared to paraffin emulsions (from 0.512 ± 0.257 μm to 0.675 ± 0.047 μm). While the droplet size for paraffin emulsions did not present statistically significant changes during storage, dodecane emulsions exhibited a significant increment in the mean particle diameter, which can be attributed to coalescence, as suggested by Zhang et al. [40]. The authors suggest that higher density oils, like liquid paraffin, reduce the buoyancy and velocity of droplets following the initial homogenization, as well as possessing greater mass per unit volume, which increases friction during droplet motion and limits the rate of coalescence and size enlargement, thus limiting emulsion destabilization [40]. These results are coherent with the EI calculations, where dodecane samples show more rapid destabilization, while paraffin samples remain stable for longer periods of time. Additionally, Ostwald ripening is unlikely to contribute to the droplet size growth considering the low solubility of dodecane in water of only 8.9 × 10^−10^ mol/mol [46].

To summarize, this study evaluates the relationship between silicon nanoparticle concentration, oil phase, and droplet size distribution in determining emulsion stability, which provides insights for various applications. The concentration of nanoparticles and oleic acid significantly affects stability, with low concentrations leading to rapid destabilization, while medium to high concentrations result in stability lasting up to 7 or 15 d. Nanoparticle modification, particularly with adsorbed oleic acid, plays a key role in enhancing stability, preventing phase separation even after an extended period, especially in emulsions with liquid paraffin. Modification of silica nanoparticles by physical adsorption of oleic acid is a simple method to enhance the nanoparticle hydrophobicity of the nanoparticles, as demonstrated in previous studies [22,47]. Consequently, the modified particles exhibit a strong affinity for the oil/water interface, facilitating the formation of stable emulsions [47]. According to Sadeghpour et al. [22], the stability of these droplets is attributed to the considerable energy required for particle detachment from the interface [22].

Additionally, droplet size distribution evidence polydispersity for both samples, with dodecane emulsions exhibiting a lower polydispersity profile. Despite some separation, stable emulsions were maintained over time, as evidenced by consistent droplet size measurements and physical stability analysis. These results offer a better understanding of the behavior of Pickering emulsions stabilized with silica nanoparticles modified with oleic acid, where the oil phases—dodecane and liquid paraffin—serve as models to evaluate the behavior of the modified nanoparticles as stabilizer. This approach opens the possibility for further studies, where the modified nanoparticles can be used to stabilize emulsions with more complex oil phases. Such systems may find applications in various fields, including pharmaceuticals, drug delivery, cosmetics, food industry, among others [20,32,48,49].

## 3. Materials and Methods

### 3.1. Materials

All aqueous solutions were prepared using distilled water. Sugarcane bagasse ash was provided by La Calandria, a panela-producing company located in San Roque, Antioquia, Colombia. Hydrochloric acid (35–37%) and sodium hydroxide (reagent grade) were obtained from Merck KGaA, Darmstadt, Germany, while dodecane, liquid paraffin, and oleic acid (>90%) were purchased from Sigma-Aldrich, St. Louis, MO, USA.

### 3.2. Nanoparticle Synthesis

**Figure 8:**
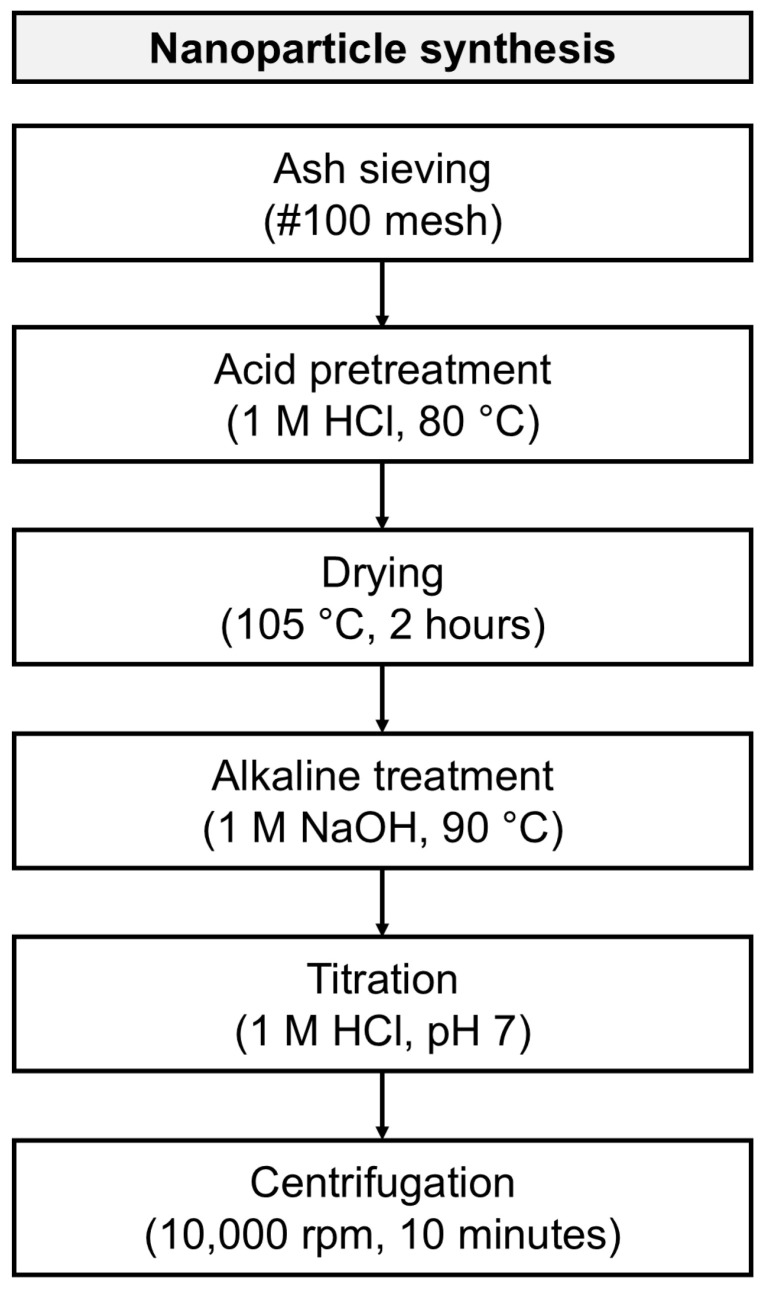
Flow diagram for silica nanoparticles production.

Synthesis of silica nanoparticles was carried out using a method based on previous works with some modifications [4,27], Figure 8 presents a scheme of the synthesis process. Initially, the raw material was sieved with a #100 mesh equivalent to an average particle size of 150 μm to achieve a uniform size distribution. Subsequently, the product underwent an acid pretreatment with 1 M hydrochloric acid (HCl) at 80 °C, continuously stirred for 1 h, with the purpose of removing metal ions and oxides. The insoluble matter was washed with deionized water until a pH of 5 was reached, then dried at 105 °C for 2 h. An ash/acid solution ratio of 1:10 by weight was used for this reaction.

In order to obtain a sodium silicate solution, the product underwent an alkaline reaction using a 1M sodium hydroxide (NaOH) solution at a 1:10 weight ratio, at 90 °C, and a continuous stirring for 1 h. The mixture was filtered, and the resulting sodium silicate solution was allowed to rest at room temperature for 24 h.

The solution was titrated with 1 M HCl, added dropwise under slow and constant stirring until reaching a pH of 7, to obtain silicon dioxide (SiO_2_). The resulting solution was aged for 24 h. After this time passed, the gel was centrifuged four times with distilled water and four times with deionized water, the supernatant was discarded to remove water-soluble impurities, using consistent centrifugation parameters (10,000 rpm for 10 min) (Hermle Z 326K, Hermle, Gosheim, Germany). Finally, the remaining solid products were stored in a refrigerator at 4 °C for subsequent characterization.

### 3.3. Adsorption Modification of Nanoparticles

Adsorption modification of the obtained nanoparticles was performed using oleic acid, based on the methodology proposed by Sadeghpour et al. [22]. The obtained nanoparticles were dispersed in distilled water using a sonic probe (Qsonica Q700, Qsonica, Newtown, CT, USA) at 20 kHz for 180 s at 30% power. Separately, oleic acid was also dispersed in distilled water by sonication for 180 s at 30% power. Subsequently, both solutions were mixed and sonicated for 180 s at 30% power to achieve the adsorption modification of silica nanoparticles.

The concentrations of nanoparticles and oleic acid were varied to study the effect of these variables on the emulsion properties. Nanoparticles concentration of 0.5 or 1 wt.% with respect to the continuous phase were chosen, while for oleic acid, 10 or 20 wt.% with respect to the quantity of nanoparticles; these conditions were selected according to the results observed in the literature [25,35,44].

### 3.4. Nanoparticles Characterization

#### 3.4.1. Field Emission Scanning Electron Microscope (FESEM)

The morphological characteristics of silica nanoparticles were examined using a field emission scanning electron microscope (FESEM) (Apreo 2 S LoVac, Thermo Fisher Scientific, Waltham, MA, USA) with a typical acceleration voltage of 20 kV and a beam current of 0.10 nA. Prior to analysis, sample dilutions were prepared following a 1:1000 ratio. A 10 μL drop of the sample was deposited onto a sample holder and allowed to rest for 24 h, to allow water evaporation at room temperature. Subsequently, the samples were coated with gold, using vacuum sputtering. Additionally, energy-dispersive X-ray spectroscopy (EDS) analysis was performed using the same equipment to study the elemental composition of the sample.

To evaluate the particle size and morphology, measurements were performed on FE-SEM micrographs using Fiji 2.16.0 [50]. Image processing included contrast enhancement, background subtraction, and threshold-based segmentation, allowing the measurement of Feret mean diameter of individual nanoparticles and aggregates. Measurements were performed on 85 particles (*n* = 85) from images at 20,000× magnification.

#### 3.4.2. Attenuated Total Reflection–Fourier Transform Infrared Spectroscopy (ATR–FTIR)

ATR-FTIR spectroscopy analysis of both modified (OA/SiO_2_ NPs) and unmodified (SiO_2_ NPs) nanoparticles was performed with an FTIR spectrometer (Thermo Scientific Nicolet iS50, Waltham, MA, USA) with a single-reflection ATR and type-IIA diamond crystal mounted on tungsten carbide. The diamond ATR had a sampling area of 0.5 mm^2^, and a consistent reproducible pressure was applied to every sample. The FTIR spectra were collected between 4000 and 400 cm^−1^ for 64 scans at a 4 cm^−1^ resolution. Each spectrum represents the average of four spectra, and all spectra were corrected using the advanced ATR correction of OMNIC 9^TM^ software (Thermo Scientific), to eliminate the diamond crystal effect and ease comparison with the transmission spectra.

### 3.5. Pickering Emulsions Fabrication

Pickering emulsions were processed, using two model oil phases, dodecane and liquid paraffin. These oil phases were selected as model phases since they are frequently used in the literature [22,35,44]. Pickering emulsions using an aqueous/oil phase ratio of 4:1 were prepared following the methodology proposed by Gao et al. [44]. The oil phase was added to the previously obtained continuous phase, and the mixture was processed using a rotor–stator homogenizer (Ultra Turrax T25, IKA, Cologne, Germany) at 16,000 rpm for 120 s. The homogenizer facilitates the dispersion of oil droplets in the continuous phase through high shear forces [49]. Subsequently, the sample was sonicated at 60% power for 240 s. This equipment induces cavitation, enabling the reduction in oil droplet size and achieving a more uniform size distribution [44].

Following the emulsification process, samples were labeled according to the type of oil phase used and the concentration of nanoparticles and oleic acid, as detailed in Table 3. Experiments were conducted in triplicate to ensure data reproducibility (n=3) and statistical analysis (Student’s *t*-test) for corresponding characterizations was performed.

### 3.6. Pickering Emulsions Characterization

#### 3.6.1. Physical Stability

The stability of all the emulsions was evaluated by visual observation with photographs on days 0, 1, 7 and 15 after their preparation [51]. Image acquisition was performed using a mobile device (iPhone 15^TM^, Apple Inc., Cupertino, CA, USA).

#### 3.6.2. Emulsification Index

The emulsification index (EI) is defined as the ratio between the height of the emulsion layer ($H_{e}$) and the total height of the sample ($H_{t}$). All samples were stored at room temperature for 15 days, and their EI were calculated using Equation (2).

(2) $$EI=\left(\frac{H_{e}}{H_{t}}\right)\times 100\%$$

#### 3.6.3. Particle Size Distribution

The size distribution of adsorption-modified SiO_2_ particles and the droplets of the obtained Pickering emulsions were determined using a static light scattering particle size analyzer (LA-960, Horiba, Kyoto, Japan) at room temperature [52]. Results were analyzed based on the average diameter (d50), volume mean diameter D4,3 as shown in Equation (3), and the relative span (δ) [53] given in Equation (4).

(3) $$D_{4,3}=\frac{\sum \left({nd}^{4}\right)}{\sum \left({nd}^{3}\right)}$$

where *n* is the number of particles with diameter *d*.

(4) $$\delta =\frac{d_{90}-d_{10}}{d_{50}}$$

where *d*_90_, *d*_10_, *d*_50_ are droplet diameters corresponding to 90%, 10%, and 50 vol.% on the accumulation curve, respectively. The relative span is employed to characterize emulsion dispersion, where values below 0.5 indicate a high degree of monodispersity [44].

## 4. Conclusions

This work proposed a green synthesis methodology for obtaining silica nanoparticles from sugarcane bagasse ash, followed by oleic acid adsorption modification to develop amphiphilic particles suitable for Pickering emulsion stabilization. The synthesized nanoparticles, primarily composed of silicon and oxygen, exhibited a degree of agglomeration, as observed through FESEM-EDS analysis. Adsorption modification with oleic acid was successfully confirmed by ATR–FTIR, revealing characteristic absorption bands of the modifier. The amphiphilic nanoparticles were then used as solid stabilizers in oil-in-water Pickering emulsions formulated with liquid paraffin and dodecane as model oil phases. The results showed that both the concentration of nanoparticles and the presence of oleic acid significantly influenced the physical stability of the emulsions, producing higher stability in emulsions formulated with liquid paraffin rather than those with dodecane. Low concentrations led to rapid destabilization, while medium to high concentrations yielded stable emulsions over 15 days in liquid paraffin emulsions. Oleic acid modification played a key role in maintaining emulsion stability, especially in formulations containing liquid paraffin.

This research contributes to the valorization of agro-industrial by-products by demonstrating that high-value materials such as silica nanoparticles can be obtained from by-products like sugarcane bagasse ash. The ash used as source material originates directly from the conventional processing of sugarcane and was used without any alteration to the existing process, enhancing the suitability of the proposed approach. The results showed that emulsions prepared with high concentrations of silica nanoparticles and oleic acid remained physically stable over time. This highlights the potential of adsorption-modified silica nanoparticles as effective stabilizers in the formulation of surfactant-free emulsions.

## Figures and Tables

**Table 1:** Effect of time on the emulsification index of emulsions with modified SiO_2_ NPs for oil phases dodecane and liquid paraffin. Values with different superscript letters within the table are significantly different (p<0.05).

Sample	Emulsification Index (EI) (%)	
	1 d	15 d
D-LM	99.092 ± 1.573 ^a^	64.125 ± 6.337 ^b^
D-LH	98.163 ± 1.597 ^a^	59.758 ± 7.735 ^b^
D-HM	99.221 ± 1.345 ^a^	66.858 ± 10.985 ^b^
D-HH	98.718 ± 1.122 ^a^	58.296 ± 1.794 ^b^
P-LM	100.000 ± 0.000 ^a^	88.638 ± 0.639 ^c^
P-LH	100.000 ± 0.000 ^a^	93.998 ± 0.923 ^d^
P-HM	100.000 ± 0.000 ^a^	90.179 ± 1.875 ^cd^
P-HH	100.000 ± 0.000 ^a^	93.828 ± 2.908 ^da^

**Table 2:** d_10_, d_50_, d_90_, D_4,3_, and δ values for emulsions with two different oil phases at different times after preparation. Values with different superscript letters in the same row are significantly different (p<0.05).

Average Diameter	Sample					
	P-HH-0d	P-HH-1d	P-HH-7d	D-HH-0d	D-HH-1d	D-HH-7d
d10 (μm)	0.197 ± 0.106 ^ab^	0.223 ± 0.012 ^a^	0.306 ± 0.075 ^ad^	0.109 ± 0.003 ^b^	0.160 ± 0.013 ^c^	0.223 ± 0.016 ^d^
d50 (μm)	0.455 ± 0.298 ^ab^	0.523 ± 0.153 ^ac^	0.643 ± 0.079 ^ad^	0.174 ± 0.006 ^b^	0.300 ± 0.035 ^c^	0.576 ± 0.042 ^d^
d90 (μm)	0.919 ± 0.312 ^ab^	0.907 ± 0.145 ^a^	1.082 ± 0.028 ^a^	0.260 ± 0.012 ^b^	0.465 ± 0.048 ^c^	0.826 ± 0.047 ^d^
4,3 (μm)	0.512 ± 0.257 ^ab^	0.546 ± 0.111 ^ac^	0.675 ± 0.047 ^a^	0.181 ± 0.007 ^b^	0.332 ± 0.014 ^c^	0.558 ± 0.039 ^d^
δ	1.822 ± 0.539 ^ab^	1.331 ± 0.116 ^a^	1.231 ± 0.292 ^ae^	0.870 ± 0.024 ^b^	1.016 ± 0.003 ^cd^	1.049 ± 0.027 ^de^

**Table 3:** Labels for the emulsions, according to their type of oil phase, SiO_2_ NPs concentration and oleic acid concentration.

Sample	Oil Phase	Silica Nanoparticles Concentration (wt.%)	Oleic Acid Concentration (wt.%)
P-LL	Liquid paraffin	0.5	0.0
P-LM	Liquid paraffin	0.5	10.0
P-LH	Liquid paraffin	0.5	20.0
P-HL	Liquid paraffin	1.0	0.0
P-HM	Liquid paraffin	1.0	10.0
P-HH	Liquid paraffin	1.0	20.0
D-LL	Dodecane	0.5	0.0
D-LM	Dodecane	0.5	10.0
D-LH	Dodecane	0.5	20.0
D-HL	Dodecane	1.0	0.0
D-HM	Dodecane	1.0	10.0
D-HH	Dodecane	1.0	20.0

## Data Availability

The datasets generated and analyzed during the current study are available from the corresponding author on reasonable request.

## Supplementary Materials

The following supporting information can be downloaded at: https://www.mdpi.com/article/10.3390/molecules30224464/s1, Figure S1: Dodecane emulsions stabilized with oleic acid at (a) 1 d and (b) 5 d; Table S1: Effect of time on the emulsification index of emulsions with modified SiO_2_ NPs for oil phases dodecane and liquid paraffin; Figure S2: Effect of time on the emulsification index for dodecane (D) and liquid paraffin (P) emulsions with different concentrations of SiO2 NPs and oleic acid as stabilizers. The graph shows that dodecane emulsions decrease their emulsification index faster than liquid paraffin emulsions, indicating that liquid paraffin emulsions are more stable over time.

## Abbreviations

The following abbreviations are used in this manuscript:

SBA	Sugarcane Bagasse Ash
SiO_2_ NPs	Silica nanoparticles
O/W	Oil/Water
W/O	Water/Oil
ATR-FTIR	Attenuated total reflection—Fourier transform infrared spectroscopy
FESEM-EDS	Field emission scanning electron microscopy and energy-dispersive X-ray spectroscopy
HCl	Hydrochloric acid
NaOH	Sodium hydroxide
