# Supplementary material for: Green Synthesis of Silica Nanoparticles from Sugarcane Bagasse Ash for Stable Pickering Oil-in-Water Emulsions
Source: Molecules. 2025 Nov 19;30(22):4464. doi: 10.3390/molecules30224464 (PMC12655093; doi:10.3390/molecules30224464)
Supplement: Supplementary file 1 [file molecules-30-04464-s001.zip › molecules-3983393-supplementary.pdf]

Supplementary Information

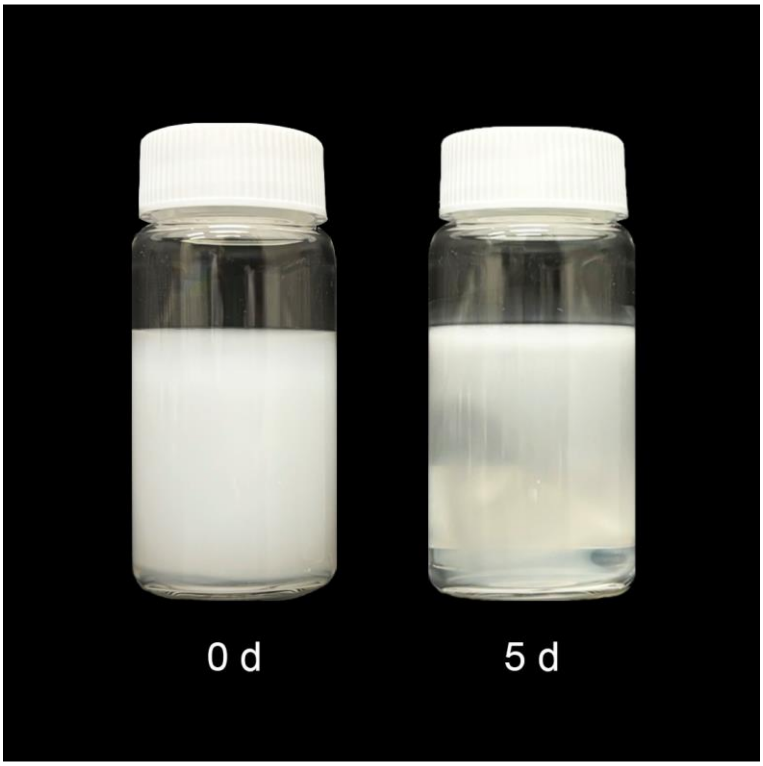

Figure S1. Dodecane emulsions stabilized with oleic acid at (a) 1 d and (b) 5 d.

Table S1. Effect of time on the emulsification index of emulsions with modified SiO_2 NPs for oil phases dodecane and liquid paraffin.

Sample	Emulsification Index (%)			
	0 d	1 d	7 d	15 d
**D-LM**	100,000 ± 0,000	99,092 ± 1,573	81,981 ± 1,261	64,125 ± 6,337
**D-LH**	100,000 ± 0,000	98,163 ± 1,597	82,354 ± 1,510	59,758 ± 7,735
**D-HM**	100,000 ± 0,000	99,221 ± 1,345	87,354 ± 6,295	66,858 ± 10,985
**D-HH**	100,000 ± 0,000	98,718 ± 1,122	89,527 ± 3,813	58,296 ± 1,794
**P-LM**	100,000 ± 0,000	100,000 ± 0,000	93,019 ± 0,947	88,638 ± 0,639
**P-LH**	100,000 ± 0,000	100,000 ± 0,000	97,774 ± 2,035	93,998 ± 0,923
**P-HM**	100,000 ± 0,000	100,000 ± 0,000	93,345 ± 1.018	90,179 ± 1,875
**P-HH**	100,000 ± 0,000	100,000 ± 0,000	96,517 ± 0,690	93,828 ± 2,908

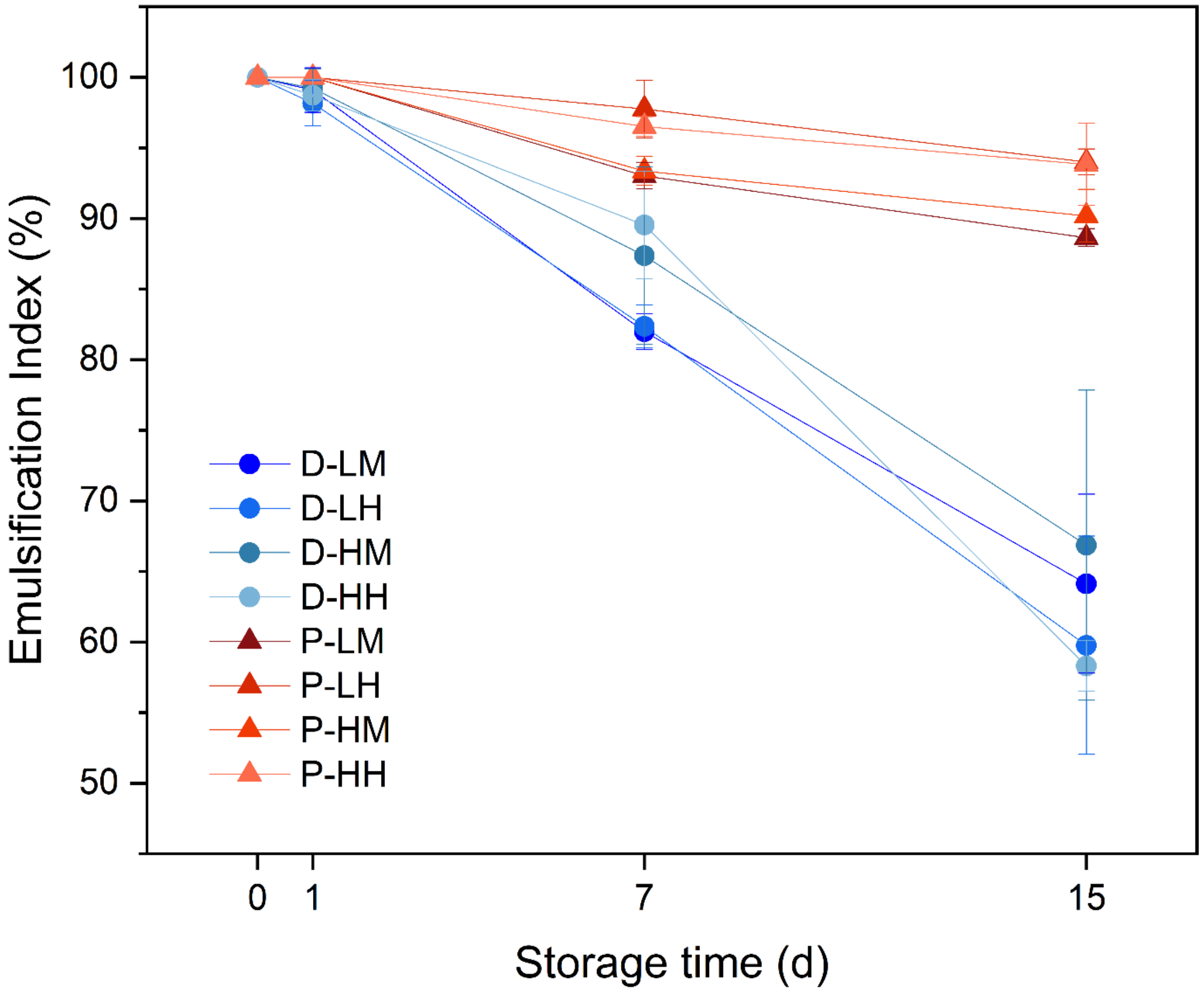

Figure S2. Effect of time on the emulsification index for dodecane (D) and liquid paraffin (P) emulsions with different concentrations of SiO2 NPs and oleic acid as stabilizers. The graph shows that dodecane emulsions decrease their emulsification index faster than liquid paraffin emulsions, indicating that liquid paraffin emulsions are more stable over time.
